# The Collateral Effect of COVID-19 on the Epidemiology of Airborne/Droplet-Transmitted Notifiable Infectious Diseases in Taiwan

**DOI:** 10.3390/antibiotics11040478

**Published:** 2022-04-03

**Authors:** Hung-Jen Tang, Chih-Cheng Lai, Chien-Ming Chao

**Affiliations:** 1 Department of Medicine, Chi Mei Medical Center, Tainan 71004, Taiwan; 8409d1@mail.chimei.org.tw; 2 Department of Internal Medicine, Kaohsiung Veterans General Hospital, Tainan Branch, Tainan 710, Taiwan; n261@mail.vhyk.gov.tw; 3 Department of Intensive Care Medicine, Chi Mei Medical Center, Liouying, Tainan 73657, Taiwan

**Keywords:** COVID-19, airborne, droplet, notifiable infectious disease, Taiwan

## Abstract

This study was conducted to compare the number of cases of airborne/droplet-transmitted notifiable infectious disease (NID) between the pandemic period (defined as from January 2020 to December 2021) and the pre-pandemic period (defined as the period from January 2018 to December 2019). The annual case numbers of airborne/droplet-transmitted NIDs from 2018 to 2021 were collected for comparison. Fourteen airborne/droplet-transmitted NIDs including measles, rubella, pertussis, influenza with severe complications, invasive pneumococcal diseases (IPD), Q fever, mumps, meningococcal meningitis, varicella, legionellosis, invasive Haemophilus influenzae type b infection, hantavirus syndrome, TB, and multidrug-resistant TB (MDRTB), were included for the analysis. Overall, the annual case number of these 14 airborne/droplet-transmitted NID was 11,930, 12,747, 9477, and 8268 in 2018, 2019, 2020, and 2021, respectively, and the overall incidence was 50.3, 53.6, 39.8, 34.6 per 100,000 populations in in 2018, 2019, 2020, and 2021. The case number of influenza with severe complications had the largest reduction from the pre-pandemic period to the pandemic period, with a reduction of 3076 cases, followed by TB (−2904), IPD (−490), mumps (−292), measles (−292), pertussis (−57), MDRTB (−43), rubella (−35), Q fever (−20), varicella (−12), meningococcal meningitis (−5), invasive *H. influenzae* type B (−4). In contrast, the case number of legionellosis increased from 492 during the pre-pandemic period to 676 during the pandemic period. In addition, hantavirus syndrome also increased from zero cases during the pre-pandemic period to three during the pandemic period. In conclusion, the occurrence of most airborne/droplet-transmitted NIDs, including both domestic and imported cases in Taiwan, was lower during the pandemic period than during the pre-pandemic period.

## 1. Introduction

Since the emergence of severe acute respiratory syndrome coronavirus 2 (SARS-CoV-2) in Wuhan, China, at the end of 2019, this novel coronavirus rapidly spread to the whole world [1]. As of 24 March 2022, there have been more than 472 million confirmed cases of coronavirus disease 2019 (COVID-19), including 6 million deaths [2]. In addition to the COVID-19 vaccine [3], every country implemented many non-pharmaceutical interventions (NPIs) to mitigate the pandemic. These NPIs included mask wearing, hand hygiene, isolation and quarantine of confirmed cases and close contacts, social distancing, contact tracing, city lockdown, border control, travel restriction, quarantine of travelers arriving from affected countries, restrictions of mass gathering, and personal protective equipment use among health workers [4,5,6]. Many NPIs have been demonstrated their effectiveness not only in the containment of COVID-19 but also help reduce several airborne/droplet-transmitted infectious diseases, such as influenza, invasive pneumococcal diseases (IPD), and measles [7,8,9,10,11,12]. In Japan, Sakamoto et al. demonstrated that seasonal influenza activity was significantly lower in 2020 than in previous years [7]. In Hong Kong, Teng et al. showed a more substantial decrease in the incidence of IPD, which is most likely attributable to the proactive mass adoption of face masks by the public [8]. In Pakistan, Rana et al. revealed a significant decline in measles cases during the COVID-19 pandemic [9].

In Taiwan, the strict implementation of NPIs helped successfully contain the outbreak of SARS-CoV-2 during this pandemic [6]. Similar to other countries, many studies showed a significant decrease in infectious disease in Taiwan after the introduction of the preventive measures against COVID-19 [13,14,15,16,17,18,19]. However, these studies compared the infectious disease in the first wave during 2020 with those during the pre-pandemic period [13,14,15,16,17,18,19]. We wonder whether this additional benefit of NPIs on non-COVID-19 infectious disease could persist for more than one year. Therefore, this study was conducted to compare the number of cases of airborne/droplet-transmitted notifiable infectious diseases (NIDs) between the pandemic period (2020 and 2021) and the pre-pandemic period (2018 and 2019).

## 2. Methods

### 2.1. The Epidemic and Control of COVID-19 in Taiwan

As of 25 March 2022, there have just been 22,463 confirmed cases of COVID-19, of which 6859 are imported, and 15,550 are domestic. The overall prevalence of COVID-19 in Taiwan was 94.0 per 100,000 populations, 853 deaths were reported, and the overall case fatality rate was 3.8% [20]. In response to the COVID-19 pandemic, Taiwanese authorities had introduced many NPIs, including universal mask wearing, hand hygiene, restrictions in crowd gathering, travel-related control, proactive screening for SARS-CoV-2, isolation of patients with COVID-19, quarantine alone or in combination with public health measures, digital contact tracing, mass vaccination, and utilization of big data technologies. The Central Epidemic Command Center was set up to allocate resources, provide health education, fight misinformation through daily press briefings, negotiate with other countries and regions, and formulate policies for mass transportation, enterprises, large-scale public gatherings, large commercial sites, community management, and the management of quarantine hotels.

### 2.2. Sources of Data

In Taiwan, a NID is any infectious disease that is required to be reported to healthcare authorities. All the associated data should be reported by clinicians or infection control nurses once a NID was suspected or diagnosed through the National Notifiable Diseases Surveillance System. Through this open database [20], everyone can freely obtain epidemiological data on NIDs. In this study, we aimed to investigate the epidemiology of airborne/droplet-transmitted NIDs, but we excluded the analysis of NIDs with zero cases during the study period from 2018 to 2022.

### 2.3. Study Period and Analysis

In this study, the annual case numbers of airborne/droplet-transmitted NIDs from 2018 to 2021 were collected for comparison. The pre-pandemic period was defined as the period from January 2018 to December 2019, and the pandemic period was defined as from January 2020 to December 2021. Percentage (%) of changes was defined as the difference in the case numbers (including both domestic and imported cases) between the pandemic period and the pre-pandemic period divided by the case numbers in the pre-pandemic period. During the study period, the population of Taiwan was 23,726,460, 23,773,876, 23,816,775, and 23,891,402 in 2018, 2019, 2020, and 2021, respectively. The annual incidence of NIDs was defined as the case number per 100,000 population.

## 3. Results

**Figure 1:**
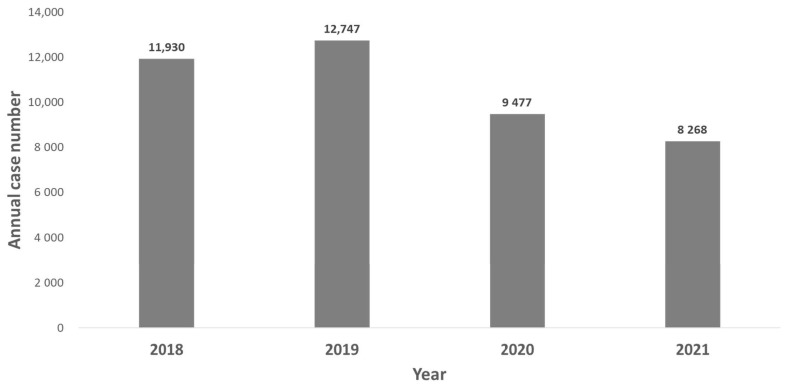
Annual case number of airborne/droplet-transmitted notifiable infectious diseases between 2018 and 2021.

**Figure 2:**
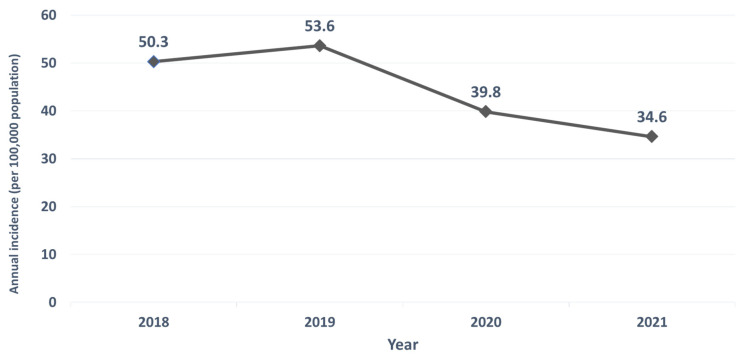
Annual incidence of airborne/droplet-transmitted notifiable infectious diseases between 2018 and 2021.

In this study, 14 airborne/droplet-transmitted NIDs including measles, rubella, pertussis, influenza with severe complications, IPD, Q fever, mumps, meningococcal meningitis, varicella, legionellosis, invasive Haemophilus influenzae type b infection, hantavirus syndrome, tuberculosis (TB), and multidrug-resistant TB (MDRTB), were included for the analysis. Overall, the annual case number of these 14 airborne/droplet-transmitted NID was 11,930, 12,747, 9477, and 8268 in 2018, 2019, 2020, and 2021, respectively (Figure 1), and the case number in the pandemic period was much lower than those in pre-pandemic period (17,775 vs. 24,677). In addition, the overall incidence was 50.3, 53.6, 39.8, 34.6 per 100,000 populations in in 2018, 2019, 2020, and 2021 (Figure 2).

### 3.1. Specific Airborne/Droplet-Transmitted NID

Table 1 summarizes the annual case number of each airborne/droplet-transmitted NID during the study period. The case number of influenza with severe complications had the largest reduction from the pre-pandemic period to the pandemic with the reduction of 3076 cases, followed by TB (−2904), IPD (−490), mumps (−292), measles (−292), pertussis (−57), MDRTB (−43), rubella (−35), Q fever (−20), varicella (−12), meningococcal meningitis (−5), invasive *H. influenzae* type B (−4). In contrast, the case number of legionellosis increased from 492 during the pre-pandemic period to 676 during the pandemic period with an increase of 184. In addition, hantavirus syndrome also increased from zero cases during the pre-pandemic period to three during the pandemic period. Moreover, the cases of measles and rubella had declined to zero during the pandemic period, with an interval change of 100%. Additionally, pertussis, influenza with severe complications, and IPD had more than a 50% of reduction during the study period.

### 3.2. Domestic Airborne/Droplet-Transmitted NID

Table 2 summarizes the annual domestic case number of each airborne/droplet-transmitted NID during the study period. The case number of influenza with severe complications had the largest reduction from the pre-pandemic period to the pandemic period with a reduction of 3065 cases, followed by IPD (−488), mumps (−279), measles (−57), pertussis (−55), Q fever (−13), varicella (−11), rubella (−8), meningococcal meningitis (−4), invasive *H. influenzae* type B (−4). In contrast, the case number of legionellosis increased from 465 during the pre-pandemic period to 6686 during the pandemic period, with an increase of 203. Moreover, the case of domestic measles and rubella had declined to zero during the pandemic period, with an interval change of 100%. Additionally, pertussis, influenza with severe complications, and IPD had more than 50% of reduction during the study period.

### 3.3. Imported Airborne/Droplet-Transmitted NID

Table 3 summarizes the annual imported case number of each airborne/droplet-transmitted NID during the study period. In addition to invasive H influenzae type B and hantavirus syndrome remaining zero during both the pre-pandemic and the pandemic period, all the other imported NIDs had lower case numbers in the pandemic period than those in the pre-pandemic period. Most of them, including measles, rubella, Q fever, pertussis, IPD, meningococcal meningitis, and varicella, had reduced to zero during the pandemic period.

## 4. Discussion

This study investigated the effect of NPIs against COVID-19 on the epidemiology of airborne/droplet-transmitted NIDs in Taiwan. We found most of these airborne/droplet-transmitted NIDs had decreased during the COVID-19 pandemic, which was based on the following evidence. First, the overall case number and the incidences of 14 NIDs were much lower during the pandemic period than those during the pre-pandemic period. Second, a similar trend—decreased case number of NIDs during the pandemic period was almost observed for each NIDs. Finally, the decreasing trend during the pandemic period remained unchanged in the subgroup analysis of domestic and imported cases. Although the causes of decreasing these NIDs during the pandemic could be multifactorial, the main explanation should be that the implementation of NPIs against COVID-19 could also help prevent the spread of these airborne/droplet-transmitted NIDs. Overall, these findings were consistent with previous studies in Taiwan [9,15,16,21,22,23] and other countries, [7,8,9,10,11] that showed that the cases of IPD, influenza, TB, and measles, significantly decreased during the pandemic. However, most of their findings were based on observations in the first wave of COVID-19. In contrast, this study compared the data in 2020 and 2021 with the corresponding period. Therefore, we could demonstrate that these collateral benefits of COVID-19 on reducing NIDs could persist for two years. Moreover, the annual case number of measles and rubella could be reduced to zero, and the case number of influenza with severe complications could be decreased to only 1 in 2021. In addition to NPIs, the enhanced and comprehensive vaccination against these NIDs during the pandemic may also contribute to these large reductions. By contrast, the vaccination against varicella is not covered by national healthcare insurance and is only recommended for patients with a high risk. Therefore, varicella had only about a 10% reduction in both overall and domestic cases in this study. All these findings indicated the persistent and collateral benefit of NPIs on the airborne/droplet-transmitted NIDs and suggested the effectiveness of NPIs.

Although this study observed that most NIDs had decreased during the pandemic, there were two exceptions—legionellosis and hantavirus syndrome. Compared with the pre-pandemic period, the case numbers of all domestic legionellosis and hantavirus infections during the pandemic period had increased in Taiwan. In contrast to hantavirus syndrome, with an increase of only three cases (from zero to three), the increase in legionella was 184 (from 492 to 676). We should be seriously concerned about this change of legionellosis. In the US, Liang et al. showed that prolonged building closures due to COVID-19 could be associated with an extreme stagnation in building water systems and further increase the presence of Legionella in the environment [24]. Another study conducted in three wards of a large regional hospital in Italy by De Giglio et al. showed similar findings that all the wards’ water networks had a higher rate of contamination by *Legionella pneumophila* (after lockdown) compared with the pre-lockdown period [25]. Despite these two observations being from other countries, it may help explain the increasing legionellosis in this study; further study investigating the prevalence of *Legionella* spp. in the water system during the pandemic is needed to clarify this issue.

This study also observed the decreasing case of imported airborne/droplet-transmitted NIDs in Taiwan. Almost all imported NIDs had reduced to zero during the pandemic. This could be due to the strict border controls in Taiwan. However, border control cannot last forever. Further surveillance investigation should be continued after the borders re-open.

In conclusion, the occurrence of airborne/droplet-transmitted NIDs in Taiwan was lower during the pandemic period than during the pre-pandemic period, which was an additional benefit of the implementation of NPIs against COVID-19. Under the continuing and evolving NPIs, these collateral benefits could persist for two years.

## Figures and Tables

**Table 1:** The case number of airborne/droplet-transmitted notifiable infectious diseases.

Disease	Case Number						Change (%)
	Years				Period		
	2018	2019	2020	2021	Pre-Pandemic Period	Pandemic Period	
Measles	40	141	0	0	181	0	−100.0
Rubella	10	25	0	0	35	0	−100.0
Pertussis	30	32	5	0	62	5	−91.9
Influenza with severe complication	1196	2325	444	1	3521	445	−87.4
Invasive pneumococcal disease	459	447	228	188	906	416	−54.1
Invasive *Haemophilus influenzae* type b infection	5	3	3	1	8	4	−50.0
Q fever	20	23	14	9	43	23	−46.5
Meningococcal meningitis	6	8	6	3	14	9	−35.7
Mumps	600	594	498	404	1194	902	−24.5
MDRTB	120	79	74	82	199	156	−21.6
TB	9179	8732	7823	7184	17,911	15,007	−16.2
Varicella	54	57	55	44	111	99	−10.8
Legionella	211	281	326	350	492	676	37.4
Hantavirus syndrome	0	0	1	2	0	3	

**Table 2:** The case number of domestic airborne/droplet-transmitted notifiable infectious diseases.

Disease	Case Number						Change (%)
	Years				Period		
	2018	2019	2020	2021	Pre-Pandemic Period	Pandemic Period	
Measles	28	82	0	0	110	0	−100.0
Rubella	1	7	0	0	8	0	−100.0
Pertussis	28	32	5	0	60	5	−91.7
Influenza with severe complication	1191	2315	440	1	3506	441	−87.4
Invasive pneumococcal disease	459	445	228	188	904	416	−54.0
Invasive *Haemophilus influenzae* type b infection	5	3	3	1	8	4	−50.0
Q fever	18	18	14	9	36	23	−36.1
Meningococcal meningitis	5	8	6	3	13	9	−30.8
Mumps	590	584	493	402	1174	895	−23.8
Varicella	54	56	55	44	110	99	−10.0
Legionella	200	265	319	349	465	668	43.7
Hantavirus syndrome	0	0	1	2	0	3	

**Table 3:** The case number of imported airborne/droplet-transmitted notifiable infectious diseases.

Disease	Case Number						Change (%)
	Years				Period		
	2018	2019	2020	2021	Pre-Pandemic Period	Pandemic Period	
Measles	12	59	0	0	71	0	−100.0
Rubella	9	18	0	0	27	0	−100.0
Q fever	2	5	0	0	7	0	−100.0
Pertussis	2	0	0	0	2	0	−100.0
Invasive pneumococcal disease	0	2	0	0	2	0	−100.0
Meningococcal meningitis	1	0	0	0	1	0	−100.0
Varicella	0	1	0	0	1	0	−100.0
Influenza with severe complication	5	10	4	0	15	4	−73.3
Legionella	11	16	7	1	27	8	−70.4
Mumps	10	10	5	2	20	7	−65.0
Invasive *Haemophilus influenzae* type b infection	0	0	0	0	0	0	
Hantavirus syndrome	0	0	0	0	0	0	
